# Recombinant Acid Ceramidase Reduces Inflammation and Infection in Cystic Fibrosis

**DOI:** 10.1164/rccm.202001-0180OC

**Published:** 2020-10-15

**Authors:** Aaron I. Gardner, Iram J. Haq, A. John Simpson, Katrin A. Becker, John Gallagher, Vinciane Saint-Criq, Bernard Verdon, Emily Mavin, Alexandra Trigg, Michael A. Gray, Albert Koulman, Melissa J. McDonnell, Andrew J. Fisher, Elizabeth L. Kramer, John P. Clancy, Christopher Ward, Edward H. Schuchman, Erich Gulbins, Malcolm Brodlie

**Affiliations:** ^1^ Translational and Clinical Research Institute, Faculty of Medical Sciences, and; ^5^ Biosciences Institute, Faculty of Medical Sciences, Newcastle University, Newcastle upon Tyne, United Kingdom; ^2^ Paediatric Respiratory Medicine, Great North Children’s Hospital, and; ^3^ Respiratory Medicine, Freeman Hospital, Newcastle upon Tyne Hospitals National Health Service Foundation Trust, Newcastle upon Tyne, United Kingdom; ^4^ Department of Molecular Biology, University of Duisburg-Essen, Essen, Germany; ^6^ National Institute for Health Research Biomedical Research Centre Metabolomics and Lipidomics Facility, University of Cambridge, Cambridge, United Kingdom; ^7^ Department of Respiratory Medicine, Galway University Hospital, Galway, Ireland; ^8^ Department of Pediatrics and; ^9^ Division of Pulmonary Medicine, Cincinnati Children’s Hospital Medical Center, Cincinnati, Ohio; ^10^ Department of Genetics and Genomic Sciences, Icahn School of Medicine at Mount Sinai, New York, New York; and; ^11^ Department of Surgery, University of Cincinnati, Cincinnati, Ohio

**Keywords:** sphingolipid, ceramide, sphingosine, lung

## Abstract

**Rationale:** In cystic fibrosis the major cause of morbidity and mortality is lung disease characterized by inflammation and infection. The influence of sphingolipid metabolism is poorly understood with a lack of studies using human airway model systems.

**Objectives:** To investigate sphingolipid metabolism in cystic fibrosis and the effects of treatment with recombinant human acid ceramidase on inflammation and infection.

**Methods:** Sphingolipids were measured using mass spectrometry in fully differentiated cultures of primary human airway epithelial cells and cocultures with *Pseudomonas aeruginosa*. *In situ* activity assays, Western blotting, and quantitative PCR were used to investigate function and expression of ceramidase and sphingomyelinase. Effects of treatment with recombinant human acid ceramidase on sphingolipid profile and inflammatory mediator production were assessed in cell cultures and murine models.

**Measurements and Main Results:** Ceramide is increased in cystic fibrosis airway epithelium owing to differential function of enzymes regulating sphingolipid metabolism. Sphingosine, a metabolite of ceramide with antimicrobial properties, is not upregulated in response to *P. aeruginosa* by cystic fibrosis airway epithelia. Tumor necrosis factor receptor 1 is increased in cystic fibrosis epithelia and activates NF-κB signaling, generating inflammation. Treatment with recombinant human acid ceramidase, to decrease ceramide, reduced both inflammatory mediator production and susceptibility to infection.

**Conclusions:** Sphingolipid metabolism is altered in airway epithelial cells cultured from people with cystic fibrosis. Treatment with recombinant acid ceramidase ameliorates the two pivotal features of cystic fibrosis lung disease, inflammation and infection, and thus represents a therapeutic approach worthy of further exploration.

**At a Glance Commentary**

**Scientific Knowledge on the Subject**

Cystic fibrosis lung disease is characterized by inflammation and susceptibility to infection. The pathogenesis of cystic fibrosis lung disease is not fully elucidated, and both of these processes remain problems in people with cystic fibrosis receiving modulator therapies. Sphingolipids form specialized membrane domains that modulate a diverse range of biological processes. Increased levels of the sphingolipid ceramide have been reported in the airway epithelium of cystic fibrosis murine models that when normalized reduced inflammation and susceptibility to the key pathogen, *Pseudomonas aeruginosa*. Studies using human model systems are lacking, representing an important gap.

**What This Study Adds to the Field**

Here, we show using differentiated cultures of airway epithelial cells from people with cystic fibrosis combined with mass spectrometry that ceramide is increased owing to differential expression and function of metabolizing enzymes. Furthermore, cystic fibrosis epithelial cells do not upregulate sphingosine, a metabolite of ceramide with antimicrobial properties, in response to *P. aeruginosa* infection. Treatment with recombinant acid ceramidase decreased ceramide levels and reduced inflammatory mediator production and susceptibility to infection in cystic fibrosis epithelial cell cultures and lung inflammation in murine models. These findings suggest a novel therapeutic approach worthy of further exploration.

Cystic fibrosis (CF) is among the most common life-limiting genetic disorders worldwide (1, 2). The major cause of morbidity and mortality in CF is lung disease characterized by neutrophilic inflammation, mucus retention, and susceptibility to endobronchial infection with, in particular, *Staphylococcus aureus* and *Pseudomonas aeruginosa* (1). A cycle of inflammation and infection ensues, resulting in progressive bronchiectasis. Several hypotheses have been proposed to explain the pathophysiology seen in CF lung disease, including abnormal volume, pH, and electrolyte content of the airway surface liquid (3–8). However, the precise mechanisms are not fully elucidated, and multiple processes that impact on inflammation and defense against infection are likely to be involved.

Sphingolipids form membrane domains that can interact to alter the function of membrane components and modulate a diverse range of biologically important processes (9). Sphingolipids have previously been linked to CF pathophysiology. Increased levels of ceramide have been identified in the airway epithelium of CF murine models that, when normalized, reduced inflammation and susceptibility to *P. aeruginosa* infection (10, 11). Raised ceramide has also been found in the epithelium of lungs removed at the time of transplantation from people with CF (12). Reports have suggested that relative concentrations of ceramides may be important, with different chain lengths over- and underrepresented in the blood of people with CF and murine models (13–15). Lipidomic analysis of BAL fluid has recently demonstrated an altered profile in CF (16, 17).

Studies using human airway model systems are lacking, representing an important gap. In part, this is because fully differentiated cultures of primary human bronchial airway epithelial cells from people with CF present technical challenges (18). In the context of sphingolipid metabolism, this human model allows investigation of underlying mechanisms and refinement of targets for intervention.

Here we investigate the metabolism of ceramides using primary human airway epithelial cell cultures, BAL fluid, and murine models. We describe how the function of key enzymes involved in ceramide metabolism—acid sphingomyelinase (which converts sphingomyelin to ceramide) and acid ceramidase (which converts ceramide to sphingosine)—is altered in CF. This promotes accumulation of ceramides that in turn leads to inflammation (generated via TNFR1 [tumor necrosis factor receptor 1] activation and NF-κB signaling) and susceptibility to infection (due to lack of upregulation of sphingosine). Treatment of airway epithelial cell cultures with recombinant human acid ceramidase (rhAC) decreased inflammation and infection. Furthermore, in murine models, nebulization of rhAC reduced airway inflammation, suggesting a therapeutic approach worthy of further investigation.

## Methods

### Primary Airway Epithelial Cell Culture and Culture Treatment

Primary bronchial epithelial cells were cultured at an air–liquid interface (ALI) as previously described (18). Clinical characteristics of the patients studied are in Table E1 in the online supplement. All cultures generated cilia, produced mucus, and had a transepithelial resistance >250 Ω · cm^2^.

For coculture experiments, ALI cultures were transitioned to antibiotic-free medium, and 1 × 10^5^ cfu of *P. aeruginosa* (PA01) in 100 μl phosphate-buffered saline were added to the apical surface and incubated for 24 hours. In specific experiments, cultures were treated apically for 1 hour with 100 μl of rhAC (20 μg/ml), generated as described previously (19); the highly selective and potent cRel inhibitor IT 901 (2 μM) for 24 hours; or the CFTR (CF transmembrane conductance regulator) modulators ivacaftor or tezacaftor–ivacaftor in combination (each 5 μM) for 48 hours with dose refreshed after 24 hours.

Detailed methods, including sample preparation for analysis, and standard methodologies (Western blotting, real-time qPCR, and ELISA) are provided in the online supplement.

### Analysis of Sphingolipid Profile of Cell Cultures by Mass Spectrometry

Calibration curves for all assayed ceramide and sphingosine species were constructed using appropriate standards. All standards and samples were analyzed in triplicate with the ABSciex QTrap 4000 system, using a 3-scan event methodology to reduce matrix noise. For selectivity, the mass tolerance for each ion was set to within 0.01 m/z, which allowed for accurate quantification.

### BAL Ceramide Measurement

For ceramide determination, a ceramide hydrolysis buffer (0.2 M citrate–phosphate buffer, 0.3 M NaCl, and 0.2 mg/ml of recombinant acid ceramidase) was mixed with the total lipid extract solution (1:1, vol/vol) and incubated at 37°C for 1 hour. Cell-free supernatant samples were analyzed using an Acquity H-Class UPLC system equipped with a Waters Acquity UPLC BEH RP18 column.

### Ceramidase and Sphingomyelinase Functional *In Situ* Assays

One hundred microliters of buffered solution containing either BODIPY TR ceramide or BODIPY FL C12-sphingomyelin at a 1:2,000 dilution was applied to the apical surface of ALI cultures. The lipid fraction was isolated as described in the online supplement, and samples were separated by thin-layer chromatography with chloroform:methanol (5:1, vol/vol).

### Mice Studies

Two different *Cftr* mutant mouse strains and their respective syngeneic littermates were used. *Cftr*^*tm1Unc*^*-Tg*^*(FABPCFTR)*^ (abbreviated *Cftr*^*KO*^) Jaw mice are genetically deficient for the murine equivalent to human CFTR (*Cftr*) but express human CFTR in the gut under control of a fatty acid–binding protein promoter to prevent acute intestinal obstruction. B6.129P2(CF/3)-*Cftr*^*TgH(neoim)Hgu*^ (abbreviated *Cftr*^*MHH*^) congenic mice that have a low residual activity of Cftr, allowing normal development and feeding, were also used.

### Nebulization of Recombinant Human Acid Ceramidase

*Cftr*^*KO*^ and *Cftr*^*MHH*^ mice were nebulized with rhAC 200 μg diluted in 800 μl 0.9% NaCl solution using a PARI BOY nebulizer apparatus over 10 minutes. rhAC was nebulized on 3 consecutive days when mice were 24 weeks old. The trachea was removed 6 hours after the last inhalation for further analysis as described in the online supplement.

## Results

### Ceramide Metabolism Is Dysregulated in Cystic Fibrosis Airway Epithelial Cells

**Figure 1.**
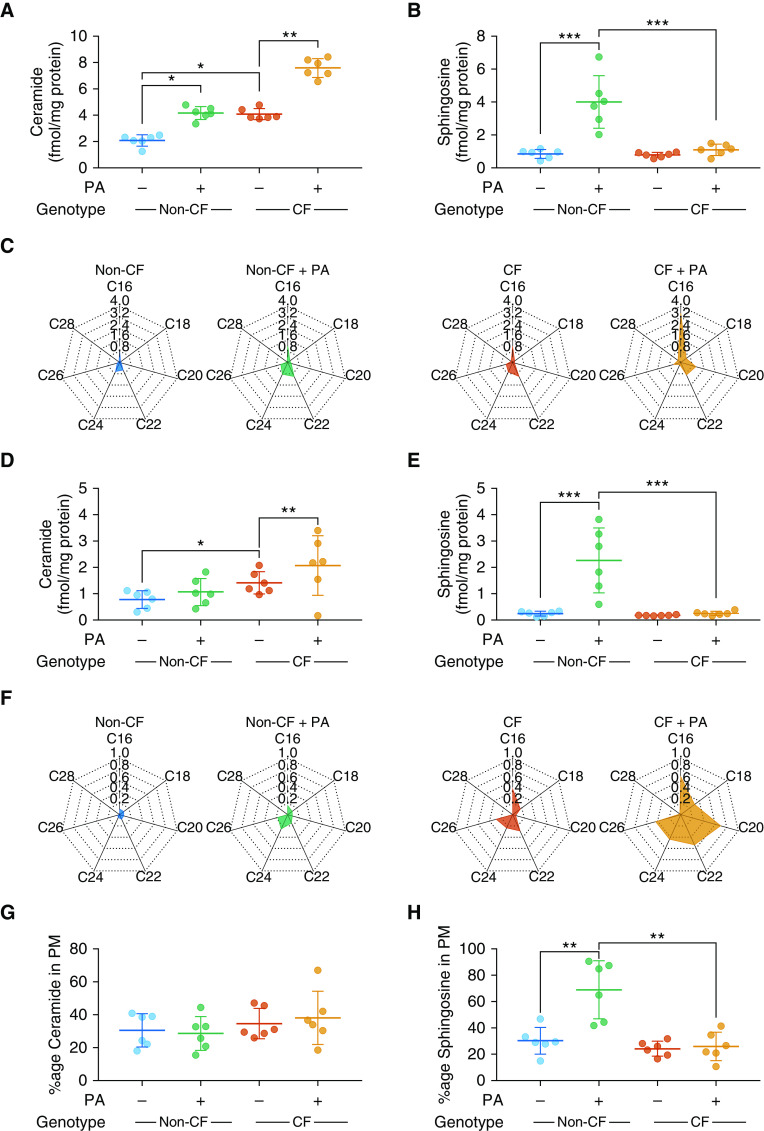
Ceramide and sphingosine levels in cystic fibrosis (CF) and non-CF fully differentiated primary human airway epithelial cell cultures at baseline and in response to *Pseudomonas aeruginosa*. (*A* and *B*) Levels of total ceramide (*A*) and sphingosine (*B*) from whole-cell lysates of CF and non-CF cultures at baseline and after coculture with *P. aeruginosa* (PA). (*C*) Radar charts of individual ceramide species (fmol/mg protein). (*D* and *E*) Plasma membrane (PM) fractions of cultures were isolated at baseline and after coculture with PA, allowing determination of total ceramide (*D*) and sphingosine (*E*) levels. (*F*) Individual ceramide species, displayed as radar charts (fmol/mg protein). (*G* and *H*) The proportion of ceramide in PMs (as a fraction of total cellular ceramide) (*G*) and equivalent for sphingosine (*H*). Throughout, *n* = 6 separate experiments from individual donors. Cultures were lysed and fractionated into whole-cell and plasma membrane fractions after 28 days at air–liquid interface and full differentiation. Individual data points are presented along with the mean (horizontal line) ± SD (error bars). For statistical tests used, *see* the online supplement. *P < 0.05, **P ≤ 0.01, and ***P ≤ 0.001.

Mass spectrometry was used to investigate the ceramide profile in primary bronchial epithelial cells isolated from people with CF or controls, and fully differentiated at an ALI. Clinical characteristics of the patients studied are in Table E1. Under basal conditions, total levels of ceramide were increased in CF cultures (Figure 1A) with similar levels of sphingosine, a key metabolite of ceramide, in CF and non-CF cultures (Figure 1B). There was increased C16 and C22 ceramide in CF cultures (Figure 1C). In murine models, normalization of sphingolipids has been associated with reduction in susceptibility to *P. aeruginosa,* the most significant respiratory pathogen in CF (10). We therefore investigated the ceramide profile of cultures after coculture with live *P. aeruginosa.* In both CF and non-CF cultures, total ceramide was increased after exposure to *P. aeruginosa* (Figure 1A), with statistically significant increases in C16, C20, and C22 observed in CF cultures only (Figure 1C). An increase in sphingosine occurred in non-CF cultures exposed to *P. aeruginosa* (Figure 1B). However, in CF cultures, *P. aeruginosa* did not induce changes. Sphingolipids are pivotal constituents of plasma membranes with enriched domains crucial for modulating cellular functions. We therefore also measured sphingolipid concentrations in plasma membrane fractions and found a similar pattern to that in whole-cell lysates for ceramide and sphingosine (Figures 1D–1F). The proportion of ceramide in membrane fractions was comparable across all experiments (Figure 1G). An increase in sphingosine occurred in non-CF cultures exposed to *P. aeruginosa* in plasma membrane fractions (Figure 1E) with an increase in percentage present in the plasma membrane (Figure 1H). Similar to results in whole-cell lysates, *P. aeruginosa* did not induce changes in plasma membrane sphingosine in CF cultures (Figures 1E and 1H).

Collectively, these data suggest that ceramide accumulates in human CF airway epithelial cells with a distinct profile of individual species present, including both long chain and very long chain ceramides. In response to *P. aeruginosa*, non-CF cultures upregulate sphingosine, an effect not seen in CF cultures.

### Ceramide Is Increased in BAL Fluid from Children and Young People with Cystic Fibrosis

**Figure 2.**
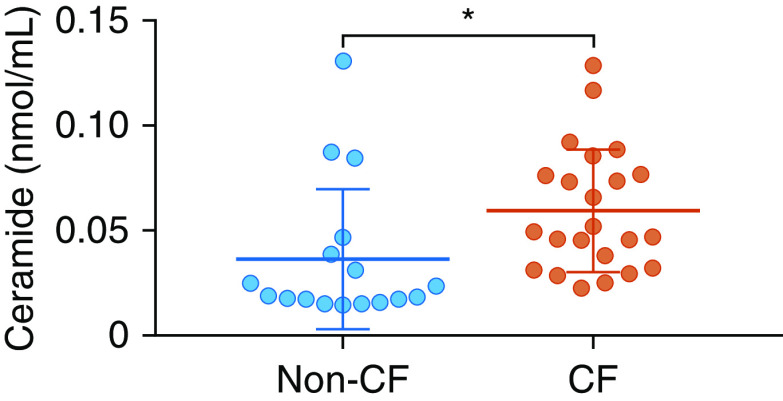
Ceramide levels in BAL fluid from children and young people. Shown are levels of ceramide in BAL fluid collected during clinically indicated bronchoscopies from children and young people with cystic fibrosis (CF) and children who do not have CF but underwent a bronchoscopy for investigation of respiratory problems. Groups were matched for age (*see* Table E1). Individual data points are presented along with the mean (horizontal line) ± SD (error bars). An unpaired *t* test was used to determine significance. *P < 0.05.

To test whether the situation *in vitro* was mirrored *in situ*, we measured total ceramide in BAL fluid collected during clinically indicated bronchoscopies from children and young people with CF and an age-matched comparator group who do not have CF but underwent investigation for respiratory problems (clinical details are in Table E1). Levels of ceramide were increased in the CF group (Figure 2).

### In Cystic Fibrosis Epithelia, There Is Decreased Function of Ceramidase and Increased Function of Sphingomyelinase

**Figure 3.**
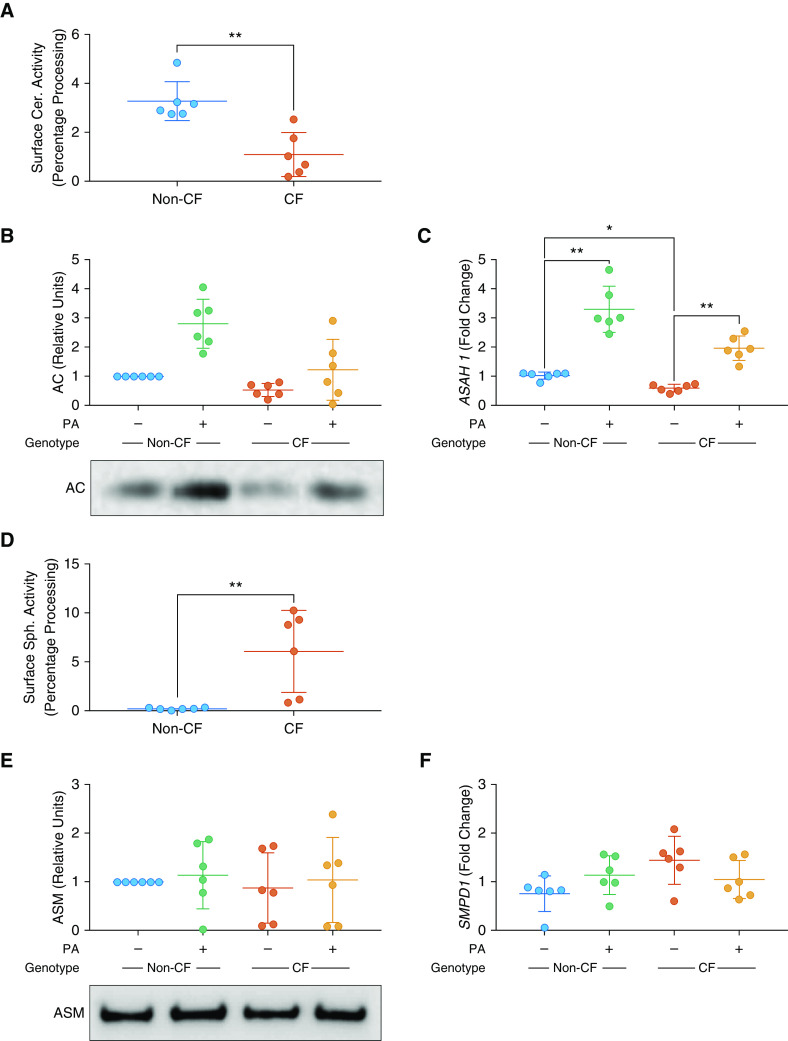
AC (acid ceramidase) and ASM (acid sphingomyelinase) expression and function in cystic fibrosis (CF) and non-CF airway epithelial cell cultures. (*A*) Apical surface activity of ceramidase, as determined by percentage of fluorescently labeled ceramide processed into sphingosine. (*B*) Levels of AC protein in CF and non-CF cultures at baseline and after coculture with *Pseudomonas aeruginosa* (PA), displayed as change relative to untreated non-CF cultures. Representative blots are shown for AC (methods for full-length blots are in the online supplement, with details of loading controls shown in Figure E1). (*C*) Gene expression of *ASAH1* (coding for acid ceramidase) at baseline in CF and non-CF cultures and after coculture with PA, displayed as fold change relative to untreated non-CF cultures. (*D*) Apical surface activity of sphingomyelinase, as determined by percentage of fluorescently labeled sphingomyelin processed into ceramide. (*E*) Levels of ASM protein, displayed as change relative to untreated non-CF cultures. Representative blots are shown for ASM (methods for full-length blots are in the online supplement, with details of loading controls shown in Figure E1). (*F*) Gene expression of *SMPD1* (coding for acid sphingomyelinase), displayed as fold change relative to untreated non-CF cultures. For loading controls, antibody, and primer and reaction details, *see* Figure E1 and Tables E2–E4. Throughout, *n* = 6 separate experiments from individual donors. Individual data points are presented along with the mean (horizontal line) ± SD (error bars). For statistical tests used, *see* the online supplement. *P < 0.05 and **P ≤ 0.01. Cer = ceramidase; Sph = sphingomyelinase.

To investigate potential mechanisms responsible for ceramide accumulation, we measured functional enzyme activity at the apical surface of cell cultures. Total ceramidase activity was reduced in CF cultures (Figure 3A). Acid ceramidase protein and *ASAH1* (coding for acid ceramidase) gene expression were decreased in CF cells compared with non-CF (Figures 3B and 3C). After coculture with *P. aeruginosa*, there was an increase in acid ceramidase protein and *ASAH1* gene expression in non-CF cultures, with a smaller increase observed in CF cultures (Figures 3B and 3C).

Conversely, sphingomyelinase activity was increased (Figure 3D) in CF cultures. However, there was no difference in acid sphingomyelinase protein or the expression of the *SMPD1* (coding for acid sphingomyelinase) gene between any cell type or treatment (Figures 3E and 3F).

Together, these data suggest that a combination of reductions in expression and function of acid ceramidase (which converts ceramide to sphingosine) and an increase in sphingomyelinase (which converts sphingomyelin to ceramide) function serve to promote the accumulation of ceramide observed in human CF airway epithelium.

### Treatment with Recombinant Human Acid Ceramidase Reduces Levels of Ceramide in Cystic Fibrosis Airway Epithelia

**Figure 4.**
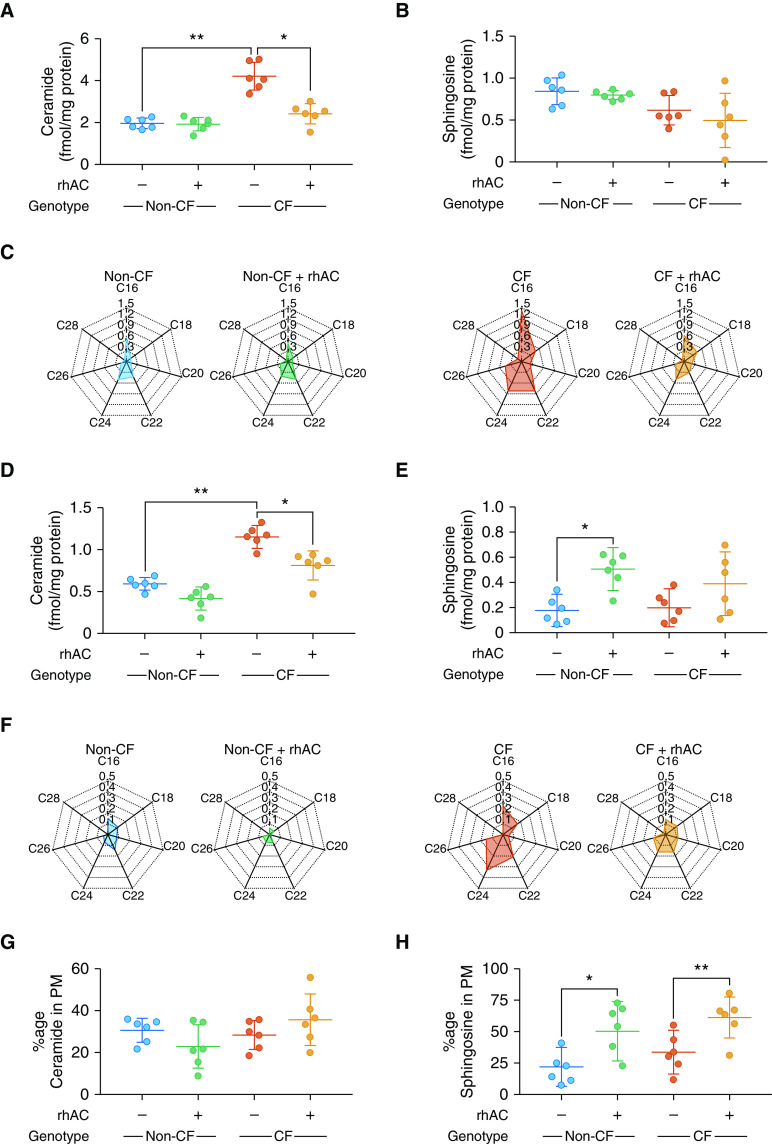
Effect of rhAC (recombinant human acid ceramidase) treatment on the ceramide and sphingosine profile of cystic fibrosis (CF) and non-CF airway epithelial cell cultures. (*A* and *B*) Levels of total ceramide (*A*) and sphingosine (*B*) from whole-cell lysates of CF and non-CF cultures at baseline and after treatment with rhAC. (*C*) Individual ceramide species, displayed as radar charts (fmol/mg protein). (*D* and *E*) Plasma membrane (PM) fractions of total ceramide (*D*) and sphingosine (*E*) in CF and non-CF cultures at baseline and after treatment with rhAC. (*F*) Individual ceramide species, displayed as radar charts (fmol/mg protein). (*G* and *H*) Proportion of ceramide in PMs (as a fraction of total cellular ceramide) (*G*) and the equivalent for sphingosine (*H*). Throughout, *n* = 6 separate experiments from individual donors. Cultures were lysed and fractionated into whole-cell and plasma membrane fractions after 28 days at air–liquid interface and full differentiation. Individual data points are presented along with the mean (horizontal line) ± SD (error bars). For statistical tests used, *see* the online supplement. *P < 0.05 and **P ≤ 0.01.

Based on Figure 3, we assessed the capacity for rhAC to modulate ceramide levels in human airway epithelial cells. rhAC has recently been developed as an enzyme replacement therapy for Farber disease (20–22). Initial experiments established no cytotoxic effect of rhAC on airway epithelial cell cultures (Figures E2A and E2B). A single treatment of CF cultures with rhAC reduced ceramide and restored levels close to those seen in non-CF cultures (Figure 4A). For sphingosine, no statistically significant differences were detected at the whole-cell level (Figure 4B). Treatment with rhAC reduced C16, C22, and C24 ceramide (Figure 4C). In plasma membrane fractions, a similar reduction in total ceramide was observed (Figure 4D) along with an increase in sphingosine levels (Figure 4E) that was statistically significant in non-CF cultures. At the individual species level, only C24 ceramide was statistically significantly reduced (Figure 4F). After rhAC treatment, the proportion of ceramide in the plasma membrane was unchanged (Figure 4G) and the proportion of sphingosine present in the plasma membrane in both CF and non-CF cultures was increased (Figure 4H).

### Inflammatory Responses Are Reduced after Treatment with Recombinant Human Acid Ceramidase

**Figure 5.**
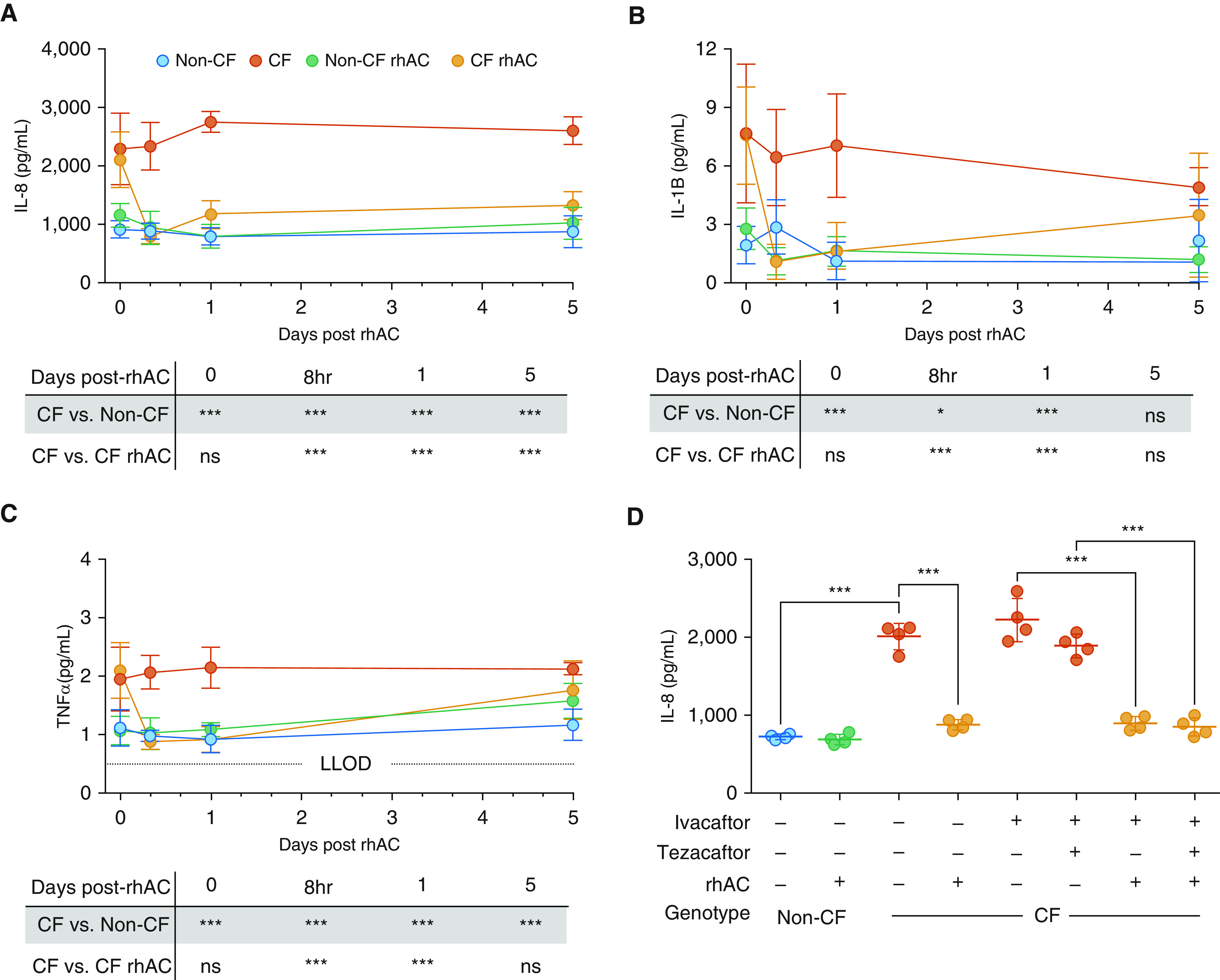
Effect of rhAC (recombinant human acid ceramidase) treatment on inflammatory mediator production by cystic fibrosis (CF) and non-CF airway epithelial cell cultures. (*A–C*) Time course of apical secretion of IL-8 (*A*), IL-1β (*B*), and TNFα (*C*) from CF and non-CF cultures at baseline and after a single treatment with rhAC. (*D*) Apical secretion of IL-8 from cultures after pretreatment with combinations of ivacaftor, tezacaftor–ivacaftor, and rhAC. (*A*–*C*) *n* = 6 separate experiments; (*D*) *n* = 4, all from individual donors. (*D*) CF group all F508del/F508del genotype. Data are presented as mean ± SD for *A*–*C*; for *D*, individual data points are presented along with the mean (horizontal line) ± SD (error bars). For statistical tests used, *see* the online supplement. *P < 0.05 and ***P ≤ 0.001. LLOD = lower limit of detection; ns = nonsignificant (*P* ≥ 0.05); TNF = tumor necrosis factor.

Neutrophilic airway inflammation is a pivotal part of CF lung disease pathophysiology (1). In view of the role of sphingolipids in modulating inflammatory responses, we investigated the effect of rhAC treatment on inflammatory mediator production at the apical surface of airway epithelial cell cultures (9). At baseline, increased secretion of IL-8, IL-1β, and TNFα (Figures 5A–5C) was observed in CF cultures, with no statistically significant differences seen in IL-4 or IL-6 (Figures E3A and E3B). Application of rhAC resulted in significantly reduced secretion of IL-8 in CF cultures, to levels comparable with control non-CF cultures, and this effect was maintained for 5 days after a single treatment (Figure 5A). A similar, though less marked, effect of rhAC was observed for IL-1β, TNFα (Figures 5B and 5C), and IL-6 (Figure E3A). Comparison was made with the effect of modulating CFTR function in CF cultures homozygous for F508del with ivacaftor or tezacaftor–ivacaftor. Again, treatment with rhAC reduced IL-8 production, with a small, but not statistically significant, reduction seen with tezacaftor–ivacaftor and no synergistic effect with rhAC and tezacaftor–ivacaftor (Figure 5D).

**Figure 6.**
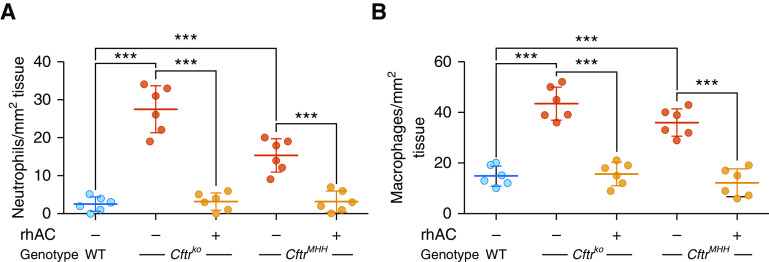
Effect of rhAC (recombinant human acid ceramidase) treatment on lung inflammation in murine models. (*A* and *B*) Number of neutrophils (*A*) and macrophages (*B*) in the submucosa of distal large bronchi in lung sections from wild-type, *Cftr*^*ko*^, and *Cftr*^*MHH*^ mice at baseline and after nebulization daily for 3 days with rhAC. Throughout, *n* = 6 mice in each group. Individual data points are presented along with the mean (horizontal line) ± SD (error bars). For statistical tests used, *see* the online supplement. ***P ≤ 0.001. WT = wild type.

To further investigate the potential for rhAC as an antiinflammatory therapy in CF, we examined the effect of nebulized rhAC on airway inflammation in two different CF murine models. *Cftr*^*tm1Unc*^*-Tg*^*(FABPCFTR)*^ (*Cftr*^*KO*^) mice are genetically deficient for the murine equivalent to human CFTR (Cftr) but express human CFTR in the gut under control of a fatty acid–binding protein promoter to prevent acute intestinal obstruction. In contrast, B6.129P2(CF/3)-*Cftr*^*TgH(neoim)Hgu*^ (*Cftr*^*MHH*^) congenic mice have a low residual activity of Cftr, allowing normal development and feeding. Increased numbers of neutrophils and macrophages were observed in the lungs of CF mice (Figures 6A and 6B). This was associated with increased ceramide levels (Figure E4A). Nebulization of rhAC to mice daily for 3 days reduced neutrophil and macrophage numbers toward wild-type levels (Figures 6A and 6B).

These data suggest that treatment with rhAC reduces the production of several key proinflammatory cytokines and chemokines by CF airway epithelial cell cultures. Furthermore, nebulization of rhAC in two different murine models was associated with a reduction in cellular markers of lung inflammation.

### Inflammatory Responses Are Driven by Altered TNFR1 Expression and NF-κB Activation

**Figure 7.**
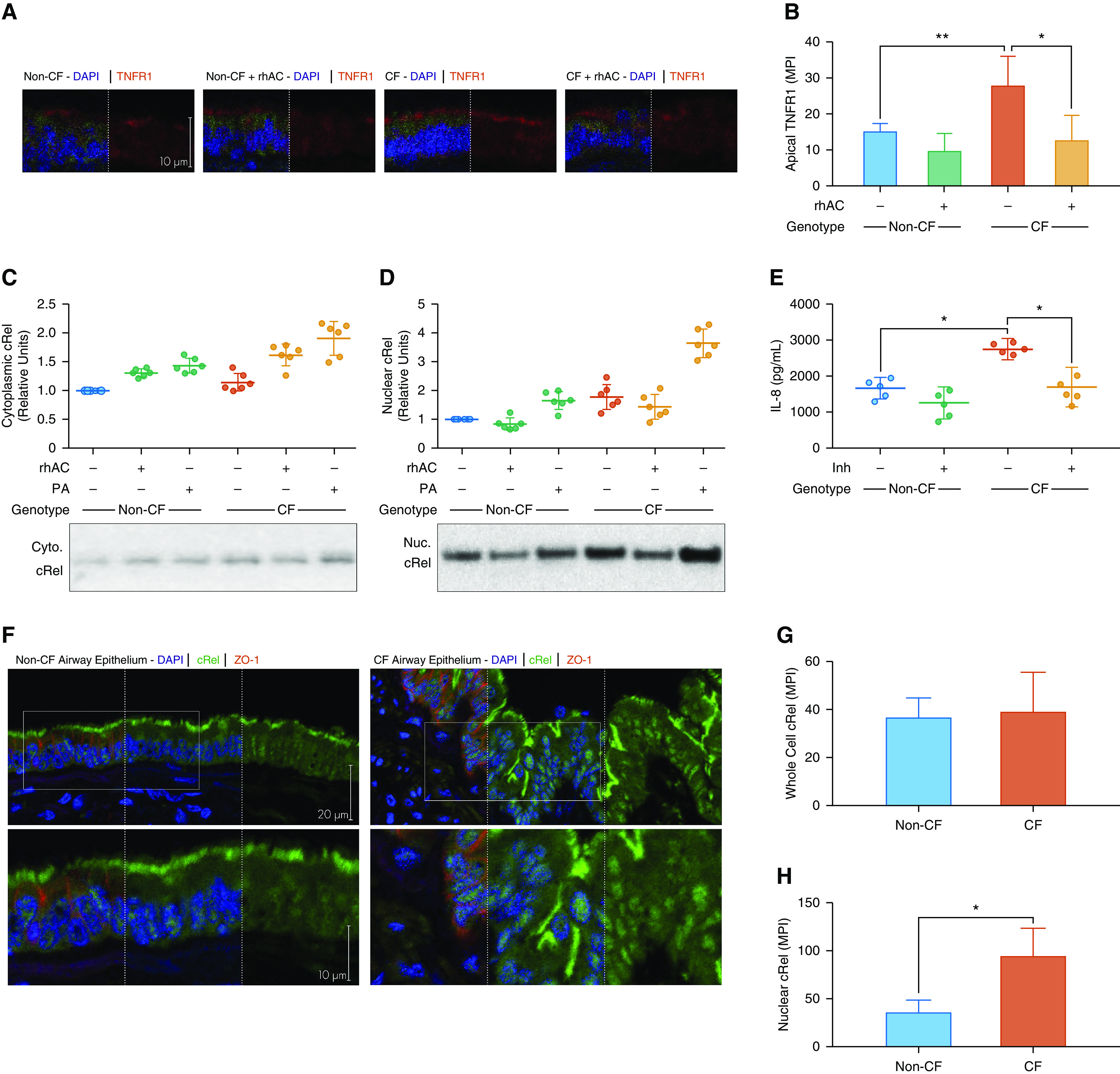
TNFR1 (tumor necrosis factor receptor 1) expression and cRel localization in cystic fibrosis (CF) airway epithelial cell cultures and lung tissue sections. (*A* and *B*) Expression of TNFR1 in CF and non-CF cultures with and without rhAC (recombinant human acid ceramidase) treatment as assessed by immunohistochemistry (*A*) with quantification of apical mean pixel intensity (*B*). (*C* and *D*) cRel expression in CF and non-CF cultures in response to *Pseudomonas aeruginosa* coculture in the presence or absence of rhAC in cytoplasmic (*C*) and nuclear (*D*) fractions. Representative blots are shown for both (methods are in the online supplement, with details of loading controls shown in Figure E1). (*E*) Apical IL-8 secretion in the presence or absence of a specific cRel inhibitor (Inh). (*F–H*) Expression and localization of cRel in airway epithelium in airway tissue sections from people with advanced CF lung disease and unused donor lungs (non-CF) (*F*) with quantification of whole-cell (*G*) and nuclear (*H*) localization. (*A*–*D*) *n* = 6 separate experiments; (*E*) *n* = 5; (*F*–*H*) *n* = 4, all from individual donors; *see* Table E1 for clinical details. Data are presented as mean ± SD for *B*, *G*, and *H*; for *C*–*E*, individual data points are presented along with the mean (horizontal line) ± SD (error bars). For statistical tests used, *see* the online supplement. *P < 0.05 and **P ≤ 0.01. Cyto. = cytoplasmic; MPI = mean pixel intensity; Nuc. = nuclear; PA = *Pseudomonas aeruginosa*.

Changes in the lipid composition of plasma membranes can significantly alter receptor expression and downstream signaling events (11, 23). Sphingolipid-enriched membrane domains have been shown to be essential for TNFR1 activation and subsequent NF-κB signaling (24). We investigated TNFR1 expression in CF cultures and found it to be increased compared with non-CF cultures (Figures 7A and 7B). This was reduced after treatment with rhAC (Figures 7A and 7B). These findings were confirmed by Western blotting for TNFR1 in plasma membrane fractions (Figure E5). To investigate downstream signaling from TNFR1, we investigated expression and nuclear localization of NF-κB subunit cRel (Figures 7C and 7D). Coculture with *P. aeruginosa* resulted in an increase in the nuclear localization of cRel in CF cultures. Treatment with rhAC increased cytoplasmic cRel, with a corresponding reduction in nuclear localization of cRel (Figures 7C and 7D). Use of a specific cRel inhibitor reduced IL-8 production (Figure 7E). In human airway tissue sections (clinical details are shown in Table E1), significantly more cRel was localized in the nucleus of epithelial cells from people with advanced CF lung disease compared with unused donor lungs (Figures 7F–7H). Due to TNFR1 also being expressed at the basolateral membrane of airway epithelial cells, we measured TNFα in the basolateral medium in the experiments shown in Figure 5C. Levels of TNFα were higher in the basolateral medium than in the apical washes (Figure E3C).

These data suggest that CF epithelia have increased TNFR1 expression that reduces after treatment with rhAC and the decrease in ceramide levels. Consequently, enhanced NF-κB activation is observed in CF epithelia with increased nuclear localization of cRel, which also reduces with rhAC treatment.

### Treatment with Recombinant Human Acid Ceramidase Reduces Susceptibility to Infection

Previous work has shown that reduction of ceramide in the airways of CF mice is associated with a reduction in susceptibility to infection (10, 11). We therefore examined effects of rhAC treatment on infection in primary human airway epithelial cells using two different methods.

**Figure 8.**
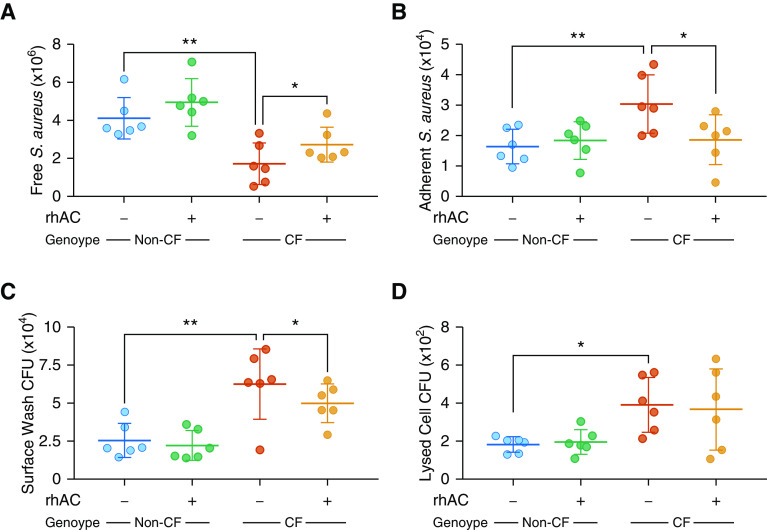
Effect of rhAC (recombinant human acid ceramidase) treatment on infection in cystic fibrosis (CF) airway epithelial cell cultures. (*A* and *B*) Number of fluorescently labeled heat-killed *Staphylococcus aureus* retrieved from apical surface washes (*A*) and adherent to apical surface (*B*) in CF and non-CF fully differentiated cultures with and without prior rhAC treatment. For representative images, *see* Figure E6 in the online supplement. (*C* and *D*) Colony-forming unit counts of *Pseudomonas aeruginosa* isolated from apical surface washes (*C*) and whole-cell lysates (*D*) (after washing, suggesting internalization) from CF and non-CF cultures with and without prior rhAC treatment. Live *P. aeruginosa* were added to the apical surface of cultures and allowed to proliferate for 24 hours. Throughout, *n* = 6 separate experiments. Individual data points are presented along with the mean (horizontal line) ± SD (error bars). For statistical tests used, *see* the online supplement. *P < 0.05 and **P ≤ 0.01.

First, heat-killed, fluorescently labeled *S. aureus* were added to the apical surface of differentiated cultures. Increased numbers of *S. aureus* remained adherent to the surface of CF cultures, suggestive of an increased susceptibility to colonization (Figures 8A and 8B). Treatment with rhAC reduced the adherence of *S. aureus* to CF cultures (Figures 8A and 8B).

Second, we investigated defense against live *P. aeruginosa.* Increased viable bacteria were isolated from the surface and lysates of CF cultures (Figures 8C and 8D). Treatment with rhAC reduced the number of viable *P. aeruginosa* recovered from the apical surface of cultures (Figure 8C), although no significant reduction in bacteria internalized by CF cultures was observed (Figure 8D).

## Discussion

Through mass spectrometry analysis of differentiated primary airway epithelial cell cultures, we have shown, and quantified, an altered ceramide profile in human CF epithelia. Increased ceramide was also detected in BAL fluid from children and young people with CF. This supports previous observations made in some murine models and agrees with immunohistochemistry performed on the bronchial epithelium of explanted advanced CF lung disease tissue (10, 12, 13).

Previous studies of sphingolipids in CF, using different models and techniques, have found varying results (15). In homogenized explanted lung tissue (containing multiple cell types and representing end-stage disease), increased C16, C18, and C20, but not C22, ceramide species were observed (12). In contrast, a cell line model transfected with an antisense *CFTR* construct demonstrated reduced levels of C18 but increased levels of C22, C24, and C26 (25). Findings in another CF murine model and the peripheral blood of people with CF have suggested a reduction in C24 ceramide and increase in C16 (13, 15). Here, using a fully differentiated model in primary human epithelial cells, we found increased C16 and C22 in whole-cell lysates and increased C24 in the plasma membrane. These ceramide chain lengths and their metabolites are known to be involved in inflammation and apoptosis (9, 12, 15, 26–31).

Data presented here suggest that ceramide accumulates in CF airway epithelia owing to differential function of enzymes. Decreased ceramidase function combined with increased sphingomyelinase activity favor increased ceramide accumulation. Western blot and gene expression analysis further showed that the decreased ceramidase activity was due to reduced acid ceramidase expression. This agrees with work in CF murine models showing that β1-integrin is trapped in the apical membrane of airway epithelial cells, downregulating acid ceramidase expression (11).

In the case of acid sphingomyelinase, we found expression to be unchanged in CF epithelia. The reason for the altered activity of acid sphingomyelinase remains uncertain. Under *in vitro* conditions, acid sphingomyelinase is a much more active enzyme than acid ceramidase (i.e., has a 10-fold or greater capacity to hydrolyze sphingomyelin compared with the ability of acid ceramidase to hydrolyze ceramide) (32). One possible explanation for reduced activity is that acid sphingomyelinase function is known to be pH dependent (activity increases as pH lowers to an optimum of around 5) (33, 34). The pH of the airway surface liquid in CF remains keenly debated; however, there is some evidence to suggest that homeostasis is disordered and that the pH is lowered (3, 4, 35–38). Another potential explanation is that acid ceramidase and acid sphingomyelinase are known to exist in a complex with interconnected functions (19). It is therefore possible that reduced acid ceramidase expression may lead to a conformational change in acid sphingomyelinase and further enhanced enzyme activity.

Treatment of CF cultures with rhAC decreased ceramide close to levels present in non-CF cultures and reduced secretion of inflammatory mediators. Nebulization of rhAC in CF mouse models also reduced lung inflammation. We found evidence of neutrophilic inflammation in the lungs of *Cftr*^*KO*^ and *Cftr*^*MHH*^ mice. A constitutive increase in expression of IL-1 and the mouse homolog of IL-8, keratinocyte-derived chemokine, has been shown in lung homogenates from these mice (10). Reduction of ceramide levels (genetically by crossing with acid sphingomyelinase knockout animals or pharmacologically via amitriptyline treatment) has also been shown to lead to a reduction in neutrophilic inflammation (10). Our work would have been strengthened by measurement of key cytokines and chemokines in the murine lung, by histological assessment of neutrophil and macrophage distribution, and by studying *in vivo* responses to airway infection.

There are divergent findings in the literature around the existence of a proinflammatory state in the CF airway in the absence of identifiable infection, with some evidence to support this concept from BAL studies in young children but varied findings in other studies and animal models (1, 39–44). In our primary human airway epithelial model, we found increased production of several proinflammatory mediators in cultures derived from adults with advanced CF lung disease. The effects of altered membrane microdomain abundance on relative fluidity and stability of transmembrane receptors involved in inflammatory responses and activity of ion channels remains poorly understood (15, 45, 46). Notably, Abu-Arish and colleagues recently demonstrated that epithelial cells respond to secretagogues by forming clusters of CFTR in ceramide-rich membrane microdomains via an acid sphingomyelinase–dependent mechanism to increase transepithelial secretion (47). We found that reducing ceramide in CF epithelial cells, via rhAC treatment, was associated with reduced TNFR1 expression, decreased cRel nuclear localization, and less IL-8 production. It is recognized that activation of NF-κB results in IL-8 production; however, epigenetic factors and mRNA stability also influence this process, and how a change in sphingolipid profile impacts on these is yet to be elucidated (48–50).

Relatively low levels of TNFα, in the range of 1–2 pg/ml in apical washings and 5–9 pg/ml in basolateral medium, were measured in unstimulated cultures, in keeping with those reported in the literature (51). However, epithelial cells are not the only source of TNFα in the CF lung, with reports of production by macrophages and neutrophils (52). Several studies have measured levels of TNFα in airway samples from people with CF. For example, mean levels of 130 and 400 pg/ml have been measured in sputum and BAL fluid, respectively, with higher concentrations during pulmonary exacerbations (52–54).

We also found increased susceptibility to infection in CF cultures that was reduced by rhAC treatment. Sphingosine, which is generated from ceramide by acid ceramidase, is known to have antimicrobial properties and has been found to be deficient in the airway of CF murine models (11). We did not detect differences in sphingosine between CF and non-CF human cultures at baseline. However, unlike non-CF cells, on exposure to *P. aeruginosa* CF epithelial cells did not upregulate levels of sphingosine in the plasma membrane. We propose that this represents a potentially important host-defense mechanism, dysfunction of which may contribute to the susceptibility to respiratory infection seen clinically in people with CF. Treatment with rhAC did increase plasma membrane sphingosine in cell cultures, but this effect was only statistically significant in non-CF cultures. A potential explanation for this is that sphingosine produced after rhAC treatment may be rapidly metabolized. In a mouse model of Farber disease, intraperitoneal rhAC treatment markedly reduced ceramide levels, but sphingosine increased to a lesser extent, especially so in the lung compartment (22). When considering the proportion of sphingosine in the plasma membrane, there was a significant increase in both CF and non-CF cultures with rhAC treatment. This localized increase in sphingosine may account for the effect of rhAC treatment on reducing bacterial adherence to the apical plasma membrane, but not internalization.

Limitations of our infection work are that we used a laboratory strain of *P. aeruginosa* rather than a clinical isolate and did not work with live *S. aureus.* Proof-of-principle data were generated to investigate the effects of acute rhAC treatment on inflammation and infection. To advance along a translational path, further evidence will likely need to be generated for longer-term treatment and efficacy in larger animal models prior to experimental medicine studies in humans.

**Figure 9.**
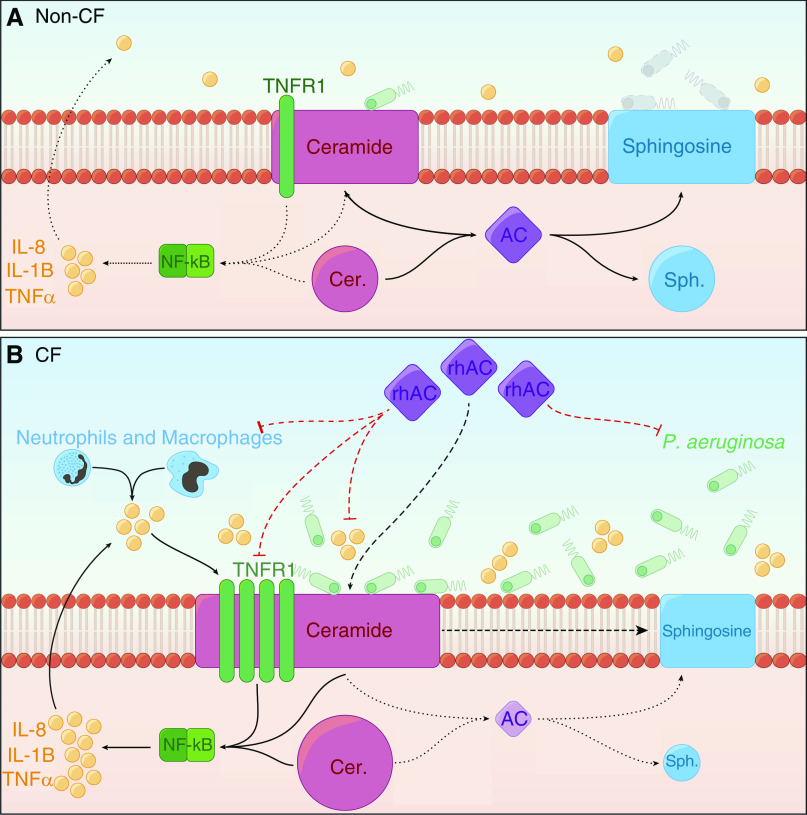
Proposed model of how altered sphingolipid metabolism in cystic fibrosis (CF) airway epithelia may result in increased inflammation and susceptibility to infection. (*A*) In non-CF epithelia, AC maintains the balance of ceramide and sphingosine. Normal levels of ceramide do not promote a proinflammatory environment, and in response to *Pseudomonas aeruginosa*, levels of sphingosine are upregulated. (*B*) In CF epithelia, AC activity is deficient in both expression and activity, which, in combination with alterations in acid sphingomyelinase activity, leads to the accumulation of ceramide. Raised ceramide is associated with increased TNFR1 expression, enhanced NF-κB activation, and nuclear localization of cRel. This promotes the secretion of proinflammatory cytokines such as IL-8, IL-1β, and TNFα. In conjunction with excessive recruitment of immune cells, which also produce proinflammatory mediators, a positive feedback loop emerges in the CF airway. CF epithelia do not upregulate sphingosine in response to *P. aeruginosa,* increasing susceptibility to infection, which further contributes to the proinflammatory environment. Treatment with rhAC reduces ceramide and increases sphingosine, ameliorating these effects. AC = acid ceramidase; Cer = ceramide; rhAC = recombinant human acid ceramidase; Sph = sphingosine; TNFR1 = tumor necrosis factor receptor 1.

Collectively, our work supports the concept that disordered sphingolipid metabolism is involved in CF lung disease pathogenesis—linking to both inflammation and infection. Our proposed model is summarized in Figure 9. Restoring acid ceramidase activity with rhAC treatment therefore represents an intriguing novel potential approach to target these two key pathological processes in the airways of people with CF. Despite exciting developments in the field of CFTR modulators, there remains an unmet need to develop therapies that ameliorate ongoing problems with inflammation and infection (55). It is also unlikely that any single medication will fully treat the complex pathophysiology of CF lung disease and highly probable that people with CF will continue to be treated with a combination of drugs in the future. The fact that rhAC is currently being developed as a treatment for patients with Farber disease highlights the potential of repurposing this drug for CF (56, 57). Toward this end, we have demonstrated that rhAC may be delivered in nebulized form to mice and has important effects *in vivo*.

## Supplementary Material

Supplements

Author disclosures
