# Supplementary material for: Recombinant Acid Ceramidase Reduces Inflammation and Infection in Cystic Fibrosis
Source: Am J Respir Crit Care Med. 2020 Oct 15;202(8):1133–45. doi: 10.1164/rccm.202001-0180OC (PMC7560813; doi:10.1164/rccm.202001-0180OC)
Supplement: Supplements [file rccm.202001-0180OC.html]

Recombinant Acid Ceramidase Reduces Inflammation and Infection in Cystic Fibrosis | American Journal of Respiratory and Critical Care Medicine

- disclosures.pdf (346 KB)
- gardner\_data\_supplement.pdf (1 MB)
