# Supplementary material for: Recombinant Acid Ceramidase Reduces Inflammation and Infection in Cystic Fibrosis
Source: Am J Respir Crit Care Med. 2020 Oct 15;202(8):1133–45. doi: 10.1164/rccm.202001-0180OC (PMC7560813; doi:10.1164/rccm.202001-0180OC)
Supplement: Supplements [file rccm.202001-0180OC_gardner_data_supplement.pdf]

Recombinant Acid Ceramidase Reduces Inflammation and Infection in Cystic Fibrosis

Online Data Supplement

Aaron I. Gardner[1], Iram J. Haq[1,2], A. John Simpson[1], Katrin A. Becker[3], John Gallagher[1], Vinciane Saint-Criq[4], Bernard Verdon[4], Emily Mavin[1], Alexandra Trigg[1], Michael A. Gray[4], Albert Koulman[5], Melissa J. McDonnell[4,6], Andrew J. Fisher[1], Elizabeth L. Kramer[7,8], John P. Clancy[7,8], Christopher Ward[1], Edward H. Schuchman[9], Erich Gulbins[3,10] and Malcolm Brodlie*[1,2]

[1]Translational and Clinical Research Institute, Faculty of Medical Sciences, Newcastle University, Newcastle upon Tyne, UK.

[2]Paediatric Respiratory Medicine, Great North Children's Hospital, Newcastle upon Tyne Hospitals NHS Foundation Trust, Newcastle upon Tyne, UK.

[3]Respiratory Medicine, Freeman Hospital, Newcastle Upon Tyne Hospitals NHS Foundation Trust, Newcastle Upon Tyne, UK

[4]Department of Molecular Biology, University of Duisburg-Essen, Essen, Germany.

[5]Biosciences Institute, Faculty of Medical Sciences, Newcastle University, Newcastle upon Tyne, UK.

[6]NIHR BRC Metabolomics and Lipidomics Facility, University of Cambridge, Cambridge, UK.

[7]Department of Respiratory Medicine, Galway University Hospital, Galway, Ireland.

[8]Department of Pediatrics, Cincinnati Children's Hospital Medical Centre, Cincinnati, USA.

[9]Division of Pulmonary Medicine, Cincinnati Children's Hospital Medical Centre, Cincinnati, USA.

[10]Department of Genetics & Genomic Sciences, Icahn School of Medicine at Mount Sinai, New York, USA.

[11]Department of Surgery, University of Cincinnati, Cincinnati, USA.

*To whom correspondence should be addressed: Malcolm Brodlie, MRC Clinician Scientist/Honorary Consultant in Paediatric Respiratory Medicine, Level 3, Clinical Resource Building, Great North Children's Hospital, Queen Victoria Road, Newcastle upon Tyne, UK, NE1 4LP. E-mail: malcolm.brodlie@ncl.ac.uk. Tel: +44 191 2336161.

Supplementary methods

Primary airway epithelial cell culture and patient demographics

Primary bronchial epithelial cells (PBECs) were isolated from the explanted native lungs of people with CF, undergoing lung transplantation. All people with CF had 2 disease-causing *CFTR* variants and had developed severe CF lung disease leading to transplantation. Patient demographics for the CF and non-CF groups are provided in Table E1. Non-CF PBECs were sampled from main bronchi via bronchoscopic brushings from 3 adults with non-CF bronchiectasis, 1 unused donor lung and 2 lobectomy specimens for distal lung cancer.

PBECs were expanded as submerged monolayer cultures, prior to differentiation as air liquid interface (ALI) cultures, as previously described(1). All cultures generated cilia, produced mucus and had a trans-epithelial resistance >250 Ω*cm^2.

Pseudomonas aeruginosa and airway epithelial cell co-cultures

One day prior to assay, ALI cultures were transitioned to completely antibiotic and serum-free medium, and washed apically. Single colonies of freshly plated *P. aeruginosa* (PA01) (ATCC) were inoculated in 10 mL of LB broth (Thermo Fisher Scientific, Waltham, MA, USA) and grown overnight at 37 °C under agitation. Optical density at 600 nM was determined and $1x10^5$ colony forming units (CFU) in 100 µL phosphate-buffered saline (PBS) were added to the apical surface of ALI cultures, and incubated for 24 hours.

Recombinant human acid ceramidase treatment of epithelial cell cultures

ALI cultures were prepared as above prior to treatment apically with 100 µL of rhAC (generated as described previously(2)) at a concentration of 20 µg/mL for 1 hour under

standard conditions. Residual liquid was then removed and cultures then either analyzed or used for ongoing culture.

Modulator treatment of epithelial cell cultures

ALI cultures treated basolaterally with either 5 μM ivacaftor or 5 μM ivacaftor and 5 μM tezacaftor (both Selleck Chemicals Houston, TX, USA), for 48 hours, with the dose replenished after 24 hours.

cRel inhibitor treatment

ALI cultures were prepared as above prior to treatment basolaterally with the highly selective and potent cRel inhibitor IT 901 (Tocris, Bristol, UK) at a final concentration of 2 μM for 24 hours under standard conditions(3).

Sample preparation

For lipid analysis

Lipids were isolated by the Folch method(4). Briefly, ALI cultures were scraped into 500 µL ice cold PBS and agitated. 10 µL was removed for BCA protein assay (Thermo Fisher Scientific), to allow for normalization, and the remainder pelleted at 5,000 x g. The pellet was resuspended in chloroform:methanol (2:1 v/v) (Sigma-Aldrich, Gillingham, UK) and gently mixed for 5 minutes. A half volume of de-ionized water was added and the sample centrifuged at 250 x g for 5 minutes to separate the phases. The lower phase was removed and evaporated at room temperature and stored at -80 °C prior to analysis. Plasma membrane fractions were isolated following a graded ultracentrifugation process.

For Western blotting

ALI cultures were scraped into 1 mL of ice cold RIPA buffer containing a protease inhibitor cocktail (Roche, Basel, Switzerland). Where required nuclear and cytoplasmic fractions were generated using the NE-PER extraction kit (Thermo Fisher Scientific). Samples were vortexed for 30 seconds before being briefly sonicated. Protein concentration was determined by BCA protein assay and samples stored at -80 °C prior to analysis.

For Real-time qPCR

ALI cultures were scraped into 500 µL ice cold PBS before being pelleted at 5,000 x g. Samples were then processed as per the Purelink RNA Micro Kit manufacturer's instructions (Thermo Fisher Scientific). RNA concentration and quality were determined using a NanoDrop One. Standard concentrations of cDNA were generated using random hexamer primers.

For cytokine quantification assay

The apical surface of cultures was washed three times with 100 µL of PBS before starting the experiment. At each timepoint the apical surface of cultures was incubated with 100 µL of PBS for 5 minutes. The fluid was aspirated and stored at -80 °C prior to analysis. 250 µL of basal media was also collected at each timepoint and replaced with fresh medium.

For immunohistochemistry

Patient tissue (clinical details are in Table E1) or ALI cultures were fixed in 4% paraformaldehyde before being dehydrated and embedded in paraffin for sequential transverse sectioning. Sections were dewaxed and rehydrated through sequential washes (Xylenes, 100%, 95%, 70%, 50% ethanol, cold tap water). Antigen retrieval consisted of boiling for 1 minute in 10 mM sodium citrate buffer. This was performed on tissue but not ALI sections.

Mass spectrometry

Calibration curves for all assayed ceramide and sphingosine species were constructed using appropriate standards (Avanti Polar Lipids, Alabaster, AL, USA). All standards and samples were analyzed in triplicate with the ABSciex QTrap 4000 system, using a 3-scan event methodology to reduce matrix noise. For selectivity the mass tolerance for each ion was set to within 0.01 m/z, which allowed for accurate quantification.

Bronchoalveolar lavage sampling and ceramide measurement

Following informed consent bronchoalveolar lavage fluid was sampled from 23 children and young people with CF (median age 8.7 years, range 0.2 to 20.8 years) and from 17 children and young people without CF (median age 8.5 years, range 0.7 to 15.2 years) but with respiratory problems undergoing clinically-indicated bronchoscopies. The physician performing the bronchoscopy determined the location of sample collection and the volume of instilled sterile saline, typically 1 to 3 instillations of 10 mL aliquots(5). Clinical characteristics are provided in Table E1. Both groups were matched for age.

Cell-free supernatant samples of bronchoalveolar lavage fluid were analyzed. For ceramide determination, a ceramide hydrolysis buffer (0.2 M citric/phosphate buffer, pH 4.5 containing 0.3 M NaCl and 0.2 mg/ml of recombinant acid ceramidase) was mixed with the total lipid extract solution (1:1,v/v) and incubated at 37 °C for 60 min. This mixture was then incubated for an additional 10 min at 50 °C with a fluorogenic reaction buffer (25 mM sodium borate buffer, pH 9) containing 1.25 mM sodium cyanide and 1.25 mM naphthalene-2,3-dicarboxyaldehyde (NDA) to derivatize the ceramide hydrolysis product, sphingosine. The mixture was then centrifuged (13,000 × g for 10 minutes) and the supernatant was analyzed using an Acquity H-Class UPLC system (Waters, Milford, MA, USA) equipped with a Waters Acquity UPLC BEH RP18 column (2.0 × 50 mm, 1.7 µm). The fluorescent (NDA) sphingosine was monitored at excitation and emission wavelengths of 252 and 483 nm, respectively. Quantification of the sphingosine peak was calculated using the Waters Empower software according to a standard curve derived from commercial (Invitrogen) NDA sphingosine.

Ceramidase and sphingomyelinase functional *in situ* assays

A derivation of the previously described fluorescent ceramidase and sphingomyelinase assays was utilized to determine enzyme activity(6). Briefly, 100 µL of buffered solution containing either BODIPY® TR Ceramide or BODIPY® FL C12-Sphingomyelin (both Thermo Fisher Scientific) at a 1:2000 dilution was applied to the apical surface of ALI cultures. After one hour of incubation residual liquid was removed and a further 2 x 100 µL PBS washes performed. The lipid fraction was isolated as described above and samples were separated by thin layer chromatography with chloroform:methanol (5:1 v/v), then analyzed on a Typhoon fluorescence plate reader.

Western blotting

20 µg of whole cell lysates, 10 µg of cytoplasmic fraction, or 6 µg of nuclear fraction, isolated as described above, were separated by SDS-PAGE and transferred to PVDF membranes (BioRad). Membranes were blocked for 1 hour with 3% BSA in PBS-Tween (Sigma) before being probed with appropriate primary and isotype-matched HRP-conjugated secondary antibodies (Table E2). Membranes were then treated with SuperSignal West PICO plus chemiluminescent substrate (Thermo Fisher Scientific) and exposed to film. Membranes were stripped and re-probed for β-actin as a loading control, and exposed. Films were scanned and samples normalized against their respective loading controls.

Real-time qPCR

Relative gene expression was determined by real-time qPCR. 1 ng of previously standardized cDNA per sample (in duplicate) was analyzed using the primers and conditions described (Tables E3 & E4) on a QuantStudio 3 system. "Template only" and "negative only" controls were performed as required, and genes of interest were normalized against combined *GAPDH, ACTB* and *TUBB*.

Cytokine quantification

Apical washes, and basal medium samples, were taken from CF and non-CF cultures which had been treated with rhAC at 0-, 8-, 24- and 120-hour intervals. Samples were analyzed using a custom Meso Scale Discovery U-PLEX assay (IL-1β, IL-4, IL-6, IL-8 and TNFα; MSD, Rockville, MD, USA), or DuoSet ELISA kit (IL-8 and TNFα; R&D Systems, Minneapolis, MN, USA) as per the manufacturer's instructions. Where

appropriate the lower limit of detection (LLOD) is displayed on the graph (any values below this threshold were excluded from statistical analysis).

Mice

Two different *Cftr* mutant mouse strains and their respective syngeneic littermates were used. $Cftr^{tm1Unc}$-$Tg^{(FABPCFTR)}$ (abbreviated $Cftr^{KO}$) Jaw mice (Jackson Laboratories, Bar Harbor, ME, US) are genetically deficient for the murine equivalent to human CFTR (*Cftr*), but express human CFTR in the gut under control of a fatty acid binding protein promoter to prevent acute intestinal obstruction(7, 8). The mice were backcrossed for more than 20 generations on a C57BL/6 background. B6.129P2(CF/3)-$Cftr^{TgH(neoim)Hgu}$ (abbreviated $Cftr^{MHH}$) congenic mice were used as previously described(9, 10). These mice have a low residual activity of Cftr allowing normal development and feeding. Mice were housed in isolator cages in a pathogen-free environment. The hygienic status was repeatedly tested by a panel of common murine pathogens according to the FELASA recommendations of 2002(11).

Nebulization of recombinant human acid ceramidase

$Cftr^{KO}$ mice were nebulized with rhAC 200 µg diluted in 800 µL 0.9% NaCl solution using Pari boy nebulizer apparatus (PARI, Starnberg, Germany) over 10 minutes. rhAC was nebulized on 3 consecutive days between 08:00 and 09:00 when mice were 24 weeks old. The trachea was removed 6 hours after the last inhalation and immediately snap frozen in liquid nitrogen.

Determination of neutrophil and macrophage numbers in murine lung tissues

Cryostat thin sections (5 µm) were prepared from lung tissues, fixed on glass slides with acetone, washed with PBS-2% Tween, blocked with normal goat serum (1:10

diluted in PBS-0.2% Tween), washed and incubated overnight at 4°C with monoclonal antibodies to murine neutrophils or to macrophages in PBS-0.5% Triton. Sections were washed with PBS-0.1% Tween and incubated with Cy2-labeled goat anti-mouse antibodies (Table E2) for 1 h at room temperature, washed 3-times with PBS-0.1% Tween and incubated with 2 µg/ml of 4,6-diamidino-2-phenylindole-dihydrochloride (DAPI; Boehringer, Mannheim, Germany) in PBS-0.1% Tween at room temperature, to allow discrimination of neutrophils and macrophages. The sections were washed and embedded with Mowiol®. For quantitative determination of cell numbers, five sections per mouse lung were examined and relevant cells were evaluated in the submucosa of distal large bronchi. The observer was blinded. From every section, 6 to 10 digitalized images were taken. Sections were analyzed with a Leica DMIRE2.

Immunohistochemistry

Slides were washed for 5 minutes in PBS plus 0.1% Triton X-100, twice, before being blocked with a 1% BSA solution in PBS-0.5% Tween for one hour at room temperature. Samples were incubated with primary antibodies (Table E2) overnight at 4 °C. Slides were washed three times for 5 minutes in PBS Tween before incubation with appropriate fluorescently conjugated secondary antibodies (Table E2) for 2 hours. Slides were washed three times for 5 minutes in PBS Tween before being incubated with DAPI as a nuclear counterstain for 5 minutes. Samples were then mounted in Mowiol® mounting media and coverslips were added. All images were acquired on a Nikon A1 confocal microscope. Appropriate blank and secondary only controls were performed as required.

Staphylococcus aureus adherence assay

100 µL of a 10,000 particles/mL solution of heat-killed, fluorescently labeled *S. aureus* particles (Sigma) were added to the apical surface of ALI cultures and incubated for 1 hour. Residual liquid was gently removed and *S. aureus* particles counted. Simultaneously, cultures were fixed in 4% PFA before being counterstained with DAPI (Sigma-Aldrich). Membranes were removed, mounted in Mowiol® mounting media and cover slips were applied. Images were acquired using a Zeiss Axioimager and counts performed with the observer blinded.

Pseudomonas aeruginosa adherence and internalization assay

P. aeruginosa-epithelial co-cultures were prepared as described above. Following rhAC treatment, 1x10^5 CFU in 100 µL of PBS were added to the apical surface of ALI cultures, and incubated for 24 hours. The apical surface of the cultures was robustly washed three times with 100 µL of PBS, and serial dilutions of the final wash were plated out for determination of adherent CFU. ALI cultures were then lysed and plated out to assess bacterial internalization.

Statistics

Mass spectrometry measurements of sphingolipids

Individual data points are presented along with the mean (horizontal line) and ± standard deviation (error bars). Statistical significance was determined using an unpaired t-test or one-way ANOVA with multiple comparisons (Tukey corrected) where appropriate. The proportion of each sphingolipid, in the plasma membrane, relative to the total amount for each sample was also calculated. Data are shown as individual points and are presented along with the mean (horizontal line) and ± standard

deviation (error bars). Statistical significance was determined using a Kruskal-Wallis test with Dunn's multiple comparisons.

Ceramidase and sphingomyelinase functional assays

Individual data points are presented along with the mean (horizontal line) and ± standard deviation (error bars). Statistical significance was determined using an unpaired t-test.

Real-time qPCR

Data are presented as fold change ($2^{-\Delta\Delta Ct}$) relative to untreated non-CF samples. Statistical significance was determined using an unpaired t-test.

Cytokine quantification

Data are presented as the mean and ± standard deviation (error bars). Statistical significance was determined using a two-way ANOVA with multiple comparisons (Tukey corrected).

Mouse immune cell counts and cytokine quantification

Individual data points are presented along with the mean (horizontal line) and ± standard deviation (error bars). Statistical significance was determined using a two-way ANOVA with multiple comparisons (Tukey corrected).

Immunohistochemistry Mean Pixel Intensity (MPI)

Individual data points are presented along with the mean (horizontal line) and ± standard deviation (error bars). Statistical significance was determined using either an unpaired t-test or one-way ANOVA with multiple comparisons (Tukey corrected).

Staphylococcus adherence assay

Individual data points are presented along with the mean (horizontal line) and ± standard deviation (error bars). Statistical significance was determined using a one-way ANOVA with multiple comparisons (Tukey corrected).

Pseudomonas aeruginosa adherence and internalization assay

Individual data points are presented along with the mean (horizontal line) and ± standard deviation (error bars). Statistical significance was determined using a one-way ANOVA with multiple comparisons (Tukey corrected).

Cell viability assays

Cells were treated with various doses of recombinant human acid ceramidase (rhAC) for 24 hours. Both the Presto Blue™ (Thermo Fisher Scientific) viability assay and lactate dehydrogenase cytotoxicity (Thermo Fisher Scientific) assay were performed as per manufacturer's instructions.

Ceramide quantification in mouse trachea

Mice were inhaled as described in the methods section. The trachea was removed after treatment, shock frozen in liquid nitrogen, homogenized in 200 µl H2O by 3 cycles of sonication using a tip sonicator and extracted in 600 µl CHCl3:CH3OH:1N HCl (100:100:1, v/v/v). The lower phase was dried, resuspended in 20 µL of a detergent solution (7.5% [w/v] n-octyl glucopyranoside, 5 mM cardiolipin in 1 mM diethylenetriaminepentaacetic acid [DTPA]), and 70 µl of 0.01 units diacylglycerol (DAG) kinase (Biomol, Germany) in 0.1 M imidazole/HCl (pH 6.6), 0.2 mM DTPA (pH 6.6), 70 mM NaCl, 17 mM MgCl2, 1.4 mM ethylene glycol tetra acetic acid; 1 µM ATP and 10 µCi [32P] ATP were added. The samples were incubated for 30 min at 37oC

with shaking (350 rpm) and processed as above. The kinase reaction was terminated by addition of 1 mL CHCl3:CH3OH:1N HCl (100:100:1, v/v/v) followed by addition of 170 µL buffered saline solution (135 mM NaCl, 1.5 mM CaCl2, 0.5 mM MgCl2, 5.6 mM glucose, 10 mM HEPES, pH 7.2) and 30 µL of a 100 mM EDTA solution were added. Phases were separated, the lower phase was collected, dried, dissolved in 20 µL of CHCl3:CH3OH (1:1, v/v) and separated on Silica G60 thin-layer chromatography (TLC) plates using CHCl3/acetone/CH3OH/acetic acid/H2O (50:20:15:10:5, v/v/v/v/v). The TLC plates were analyzed employing a phospho-imager. Ceramide was determined by comparison with a standard curve of C16- to C24-ceramides.

Study approvals

Written informed consent was received from participants prior to inclusion. The use of tissue and cells was approved by Local Research Ethics Committees (Newcastle and North Tyneside, reference 11/NE/029 and Galway University Hospitals, reference C.A.771). Bronchoalveolar lavage sampling was approved by the Cincinnati Children's Hospital Medical Center Institutional Review Board. All procedures performed on mice were approved by the Animal Care and Use Committee of the Bezirksregierung Duesseldorf, Duesseldorf, Germany.

Supplementary figures

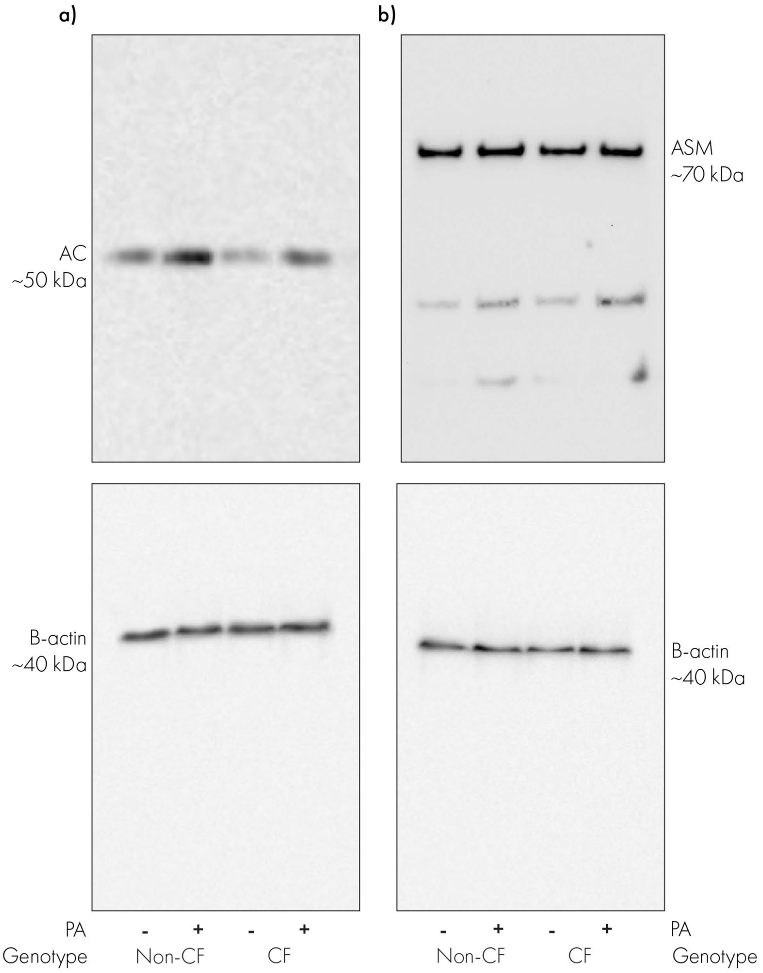

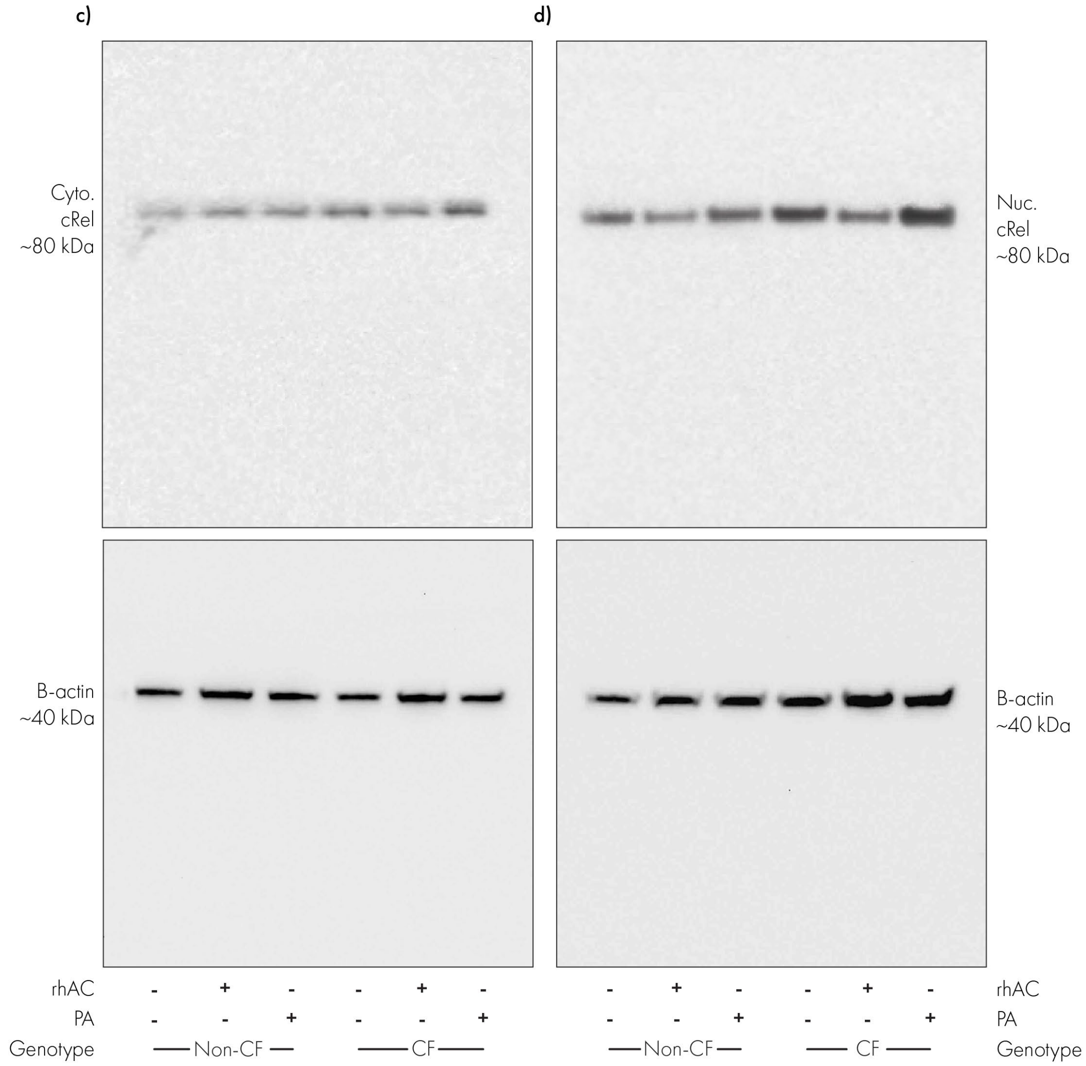

Figure E1 – Representative full-length Western blots and loading controls for acid ceramidase, acid sphingomyelinase and cRel expression in cystic fibrosis and non-cystic fibrosis epithelial cell cultures from the main manuscript. Corresponding to Figs 3b, 3e, 7c and 7d in the main manuscript. Representative full-length Western blots for acid ceramidase (AC) (a), acid sphingomyelinase (ASM) (b), cytoplasmic cRel (c) and nuclear cRel (d), with associated β-actin loading controls from the same membrane.

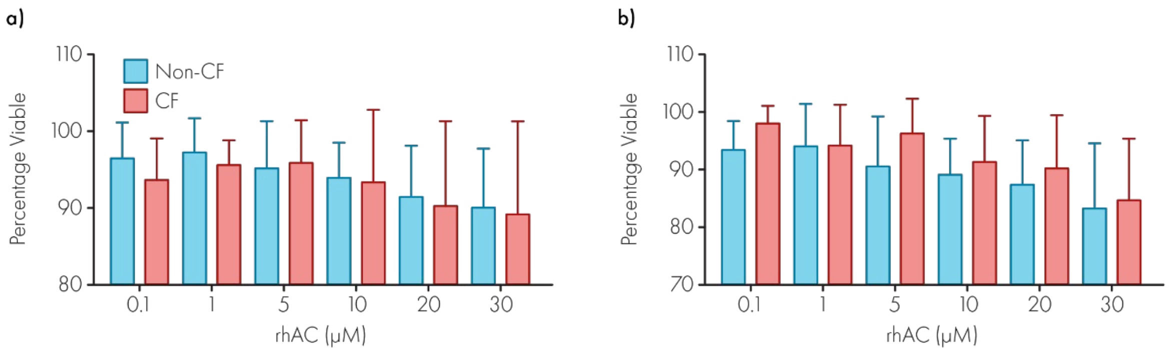

Figure E2 – Recombinant human acid ceramidase toxicity studies in airway epithelial cells. Effect of differing concentrations of recombinant human acid ceramidase (rhAC) on cell viability of cystic fibrosis (CF) and non-CF airway epithelial cell cultures measured by (a) Presto Blue™ viability assay and (b) lactate dehydrogenase cytotoxicity assay. Throughout, n=5 individual donors. Data are presented as mean with standard deviation. For statistical tests used see methods. No significant differences detected.

a)

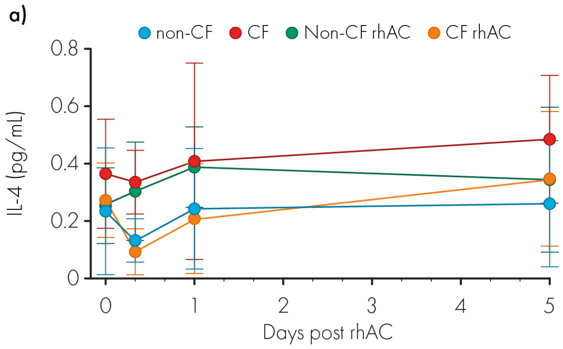

Days post rhAC	0	8hr	1	5
CF vs Non-CF	ns	ns	ns	ns
CF vs CF rhAC	ns	ns	ns	ns

b)

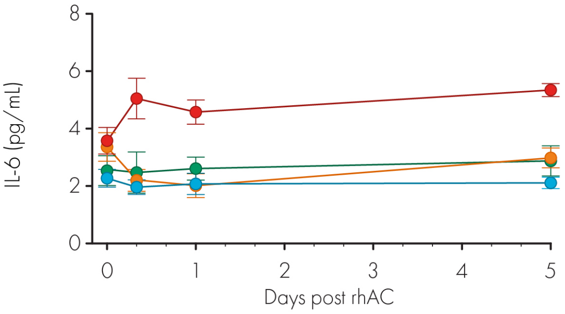

Days post rhAC	0	8hr	1	5
CF vs Non-CF	ns	***	***	***
CF vs CF rhAC	ns	***	***	**

c)

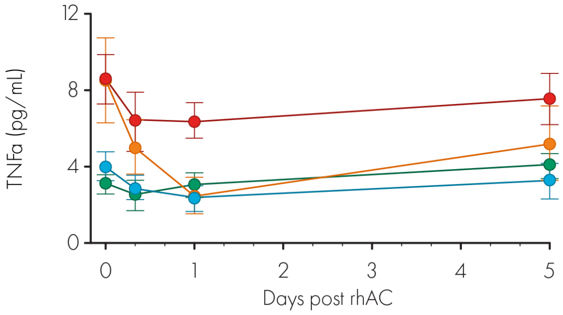

Days post rhAC	0	8hr	1	5
CF vs Non-CF	***	***	***	***
CF vs CF rhAC	ns	ns	**	ns

Figure E3 - Effect of recombinant human acid ceramidase treatment on inflammatory mediator production by cystic fibrosis and non-cystic fibrosis airway epithelial cell cultures (other cytokines and chemokines not shown in Figure 5). The apical surface of cystic fibrosis (CF) and non-CF airway epithelial cell cultures was washed with 100 µL sterile phosphate buffered saline. Cultures were then apically treated with recombinant human acid ceramidase (rhAC) and additional washes performed at 8-, 24- and 120-hours post-treatment (a) IL-4 and (b) IL-6 apical secretion time course. (c) TNFα measured in the basolateral medium over the same experiments. LLOD refers to lower limit of detection for assay used. Throughout n=6 individual donors. Data presented as mean with standard deviation. For statistical tests used see methods ***P*≤0.01, ****P*≤0.001 and ns non-significant *P*≥0.05.

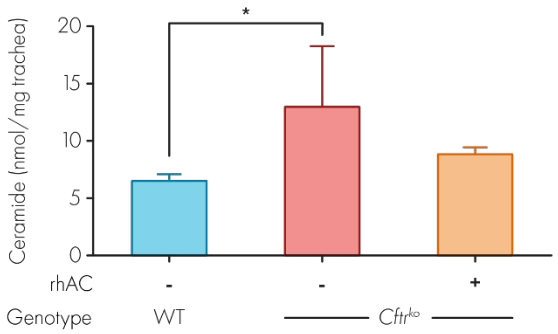

Figure E4 – Effect of recombinant human acid ceramidase treatment on ceramide concentration in the trachea of mice. (a) Concentration of ceramide in the trachea of wild type (WT) and *Cftr*^{KO} mice at baseline and following daily nebulization for 3 days of recombinant human acid ceramidase (rhAC). Throughout, n=4 mice. Data presented as mean with standard deviation. For statistical tests used see methods *P<0.05.

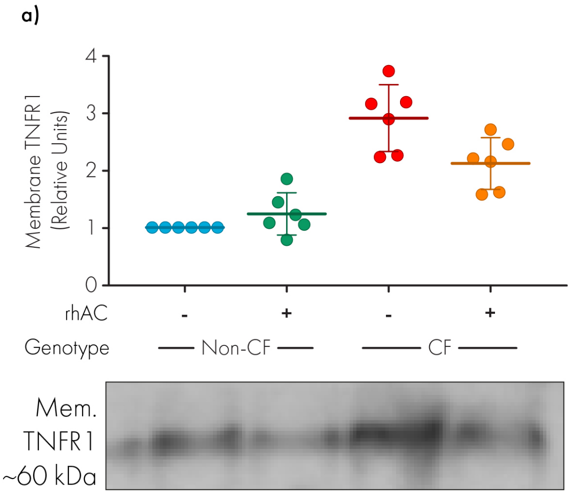

Figure E5 – Western blotting of Tumor Necrosis Factor Receptor 1 in the plasma membrane of cystic fibrosis and non-cystic fibrosis airway epithelial cell cultures. Representative Western blot and quantification of Tumor Necrosis Factor Receptor 1 (TNFR1) present in the plasma membrane fraction of cystic fibrosis (CF) and non-CF airway epithelial cultures. Membrane fractions from standardized whole cell lysates were separated through the use of ultracentrifugation prior to Western blotting. Samples were standardized against a non-CF control as no reliable housekeeping marker could be determined for the plasma membrane fraction. Throughout n=6 individual donors. Individual data points are presented along with the mean (horizontal line) and ± standard deviation (error bars).

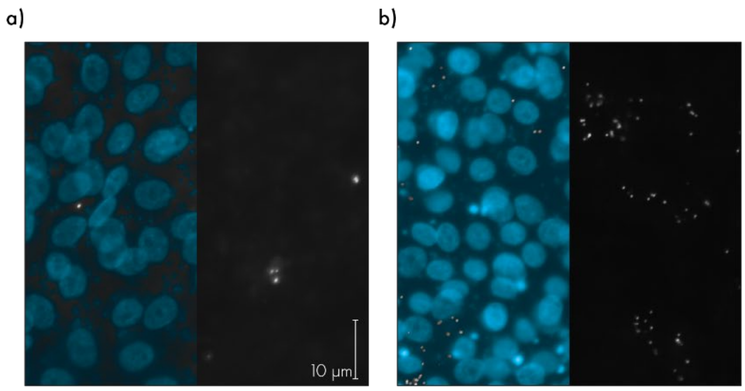

Figure E6 – Representative images of *Staphylococcus aureus* particles adherent to the surface of non-cystic fibrosis and cystic fibrosis airway epithelial cell cultures. Representative images of fluorescently labeled *Staphylococcus aureus* adherent to the surface of (a) non-cystic fibrosis (CF) and (b) CF fully differentiated airway epithelial cell cultures. Fluorescent particles are visible as white dots in the merge (left) and without DAPI (right) panels.

Supplementary tables

Table E1 – Demographics of patients sampled in the study

Diagnosis	*CFTR* genotype	Age (years)	Sex
**Demographics of people sampled for primary airway epithelial cell culture (Figures 1,3,4,5,7,8)**			
Cystic fibrosis	p.Phe508del / p.Arg560Thr	43	Male
	p.Phe508del / p.Phe508del	41	Male
	p.Phe508del / p.Phe508del	30	Male
	p.Phe508del / p.Phe508del	36	Male
	p.Phe508del / p.Phe508del	28	Female
	p.Phe508del / p.Gly542Ter	32	Male
Non-CF bronchiectasis	*Not applicable*	34	Female
Non-CF bronchiectasis		47	Female
Non-CF bronchiectasis		34	Male
Unused donor lung		28	Male
Lobectomy		44	Female
Lobectomy		48	Male
**Demographics of children and young people from whom bronchoalveolar lavage fluid was sampled (Figure 2)**			
Cystic fibrosis	Not recorded	18	Female
	p.Phe508del / p.Phe508del	3	Female
	p.Phe508del / p.Leu206Trp	2 months	Female
	p.Phe508del / p.Phe508del	3 months	Male
	p.Phe508del / p.Phe508del	1	Male
	p.Phe508del / p.Phe508del	4	Female
	c.1022_1023insTC / Unknown	5	Male
	p.Phe508del / p.Phe508del	7	Female
	p.Phe508del / p.Phe508del	16	Male
	p.Phe508del / p.Phe508del	7	Female
	p.Phe508del / p.Phe508del	6	Male
	p.Phe508del / c.3272-76A>G	16	Female
	p.Phe508del / c.489+1G>T	10	Female
	Not recorded	13	Female
	p.Phe508del / p.Phe508del	12	Female
	Not recorded	17	Male
	p.Phe508del / p.Phe508del	8	Female
	Not recorded	15	Male
	p.Phe508del / p.Phe508del	11	Female
	p.Phe508del / p.Phe508del	6	Male
	p.Phe508del / p.Phe508del	3	Male
	Not recorded	15	Female
	Not recorded	20	Male
Upper airway obstruction	*Not applicable*	8 months	Male
Tracheomalacia		1	Male
Not recorded		2	Male
Subglottic stenosis		3	Male
Subglottic stenosis		3	Male

Diagnosis	*CFTR* genotype	Age (years)	Sex
Subglottic stenosis, bronchiectasis	*Not applicable*	3	Female
Tracheomalacia		6	Male
Adenotonsillar hypertrophy		6	Male
Upper airway obstruction		8	Female
Bronchiectasis		9	Female
Recurrent pneumonia		11	Female
Upper airway obstruction		13	Female
Not recorded		13	Male
Subglottic stenosis, bronchiectasis		15	Male
Not recorded		15	Female
Upper airway obstruction, bronchiectasis		17	Male
Tracheal stenosis		18	Male
**Demographics of patients sampled for immunohistochemistry of lung tissue (Figure 7)**			
Cystic fibrosis	p.Phe508del / p.Phe508del	41	Male
	p.Phe508del / p.Phe508del	30	Male
	p.Phe508del / p.Phe508del	36	Male
	p.Phe508del / p.Phe508del	28	Female
Unused donor lung	*Not applicable*	52	Female
Unused donor lung		61	Female
Unused donor lung		52	Female
Unused donor lung		64	Male

Table E2 – Antibody details

Target	Host (isotype)	Supplier	Catalogue number
Acid ceramidase	Rabbit (IgG)	Mybiosource	MBS1492517
Acid sphingomyelinase	Mouse (IgG)	Abcam	ab74281
CD68	Mouse (IgG1)	Acris	AM50195PU
TNFR1	Rabbit (IgG)	Abcam	ab19139
cRel	Rabbit (IgG)	Cell Signaling	4727S
Actin	Mouse (IgG2a)	Sigma	A2228
Cy2 conjugated anti mouse	Goat	Abcam	ab6944
HRP conjugated anti mouse	Goat	ThermoFisher Scientific	31430
HRP conjugated anti rabbit	Goat	ThermoFisher Scientific	31460
AF488 conjugated anti mouse	Goat	ThermoFisher Scientific	A-11001
AF488 conjugated anti rabbit	Goat	ThermoFisher Scientific	A-11008
AF594 conjugated anti mouse	Goat	ThermoFisher Scientific	A-11005
AF594 conjugated anti rabbit	Goat	ThermoFisher Scientific	A-11012

Table E3 – qPCR reaction details

	UDG activation	Denature	Denature	Anneal/Extend
**Temperature**	50 °C	95 °C	95 °C	60 °C
**Time**	2 min	10 min	15 sec	1 min

Table E4 – qPCR primer details

Gene	Forward Primer	Reverse Primer
ASAH1	ACCAGTGCCTGGCCTACTT	AACAGCGGCAATACCCTTCA
SMPD1	GAAGGGAAAAGAAAGAATTGGGGG	GAGAGAGATGAGGCGGAGAC
GAPDH	GTCTCCTCTGACTTCAA	ACCACCCTGTTGCTGTA
ACTB	TGAGAGGGAAATCGTGCGTG	TGCTTGCTGATCCACATCTGC
TUBB	ACTACCAGCCACCCTCTGTGTC	GCACAAACGCACGATTACA

Supplementary references

E1. Brodlie M, McKean MC, Johnson GE, Perry JD, Nicholson A, Verdon B, Gray MA, Dark JH, Pearson JP, Fisher AJ, Corris PA, Lordan J, Ward C. Primary bronchial epithelial cell culture from explanted cystic fibrosis lungs. *Exp Lung Res* 2010; 36: 101-110.

E2. He X, Okino N, Dhami R, Dagan A, Gatt S, Schulze H, Sandhoff K, Schuchman EH. Purification and characterization of recombinant, human acid ceramidase. Catalytic reactions and interactions with acid sphingomyelinase. *J Biol Chem* 2003; 278: 32978-32986.

E3. Shono Y, Tuckett AZ, Liou HC, Doubrovina E, Derenzini E, Ouk S, Tsai JJ, Smith OM, Levy ER, Kreines FM, Ziegler CG, Scallion MI, Doubrovin M, Heller G, Younes A, O'Reilly RJ, van den Brink MR, Zakrzewski JL. Characterization of a c-Rel Inhibitor That Mediates Anticancer Properties in Hematologic Malignancies by Blocking NF-kappaB-Controlled Oxidative Stress Responses. *Cancer Res* 2016; 76: 377-389.

E4. Folch J, Lees M, Sloane Stanley GH. A simple method for the isolation and purification of total lipides from animal tissues. *J Biol Chem* 1957; 226: 497-509.

E5. Noah TL, Black HR, Cheng PW, Wood RE, Leigh MW. Nasal and bronchoalveolar lavage fluid cytokines in early cystic fibrosis. *J Infect Dis* 1997; 175: 638-647.

E6. Grassme H, Henry B, Ziobro R, Becker KA, Riethmuller J, Gardner A, Seitz AP, Steinmann J, Lang S, Ward C, Schuchman EH, Caldwell CC, Kamler M, Edwards MJ, Brodlie M, Gulbins E. beta1-Integrin Accumulates in Cystic Fibrosis Luminal Airway Epithelial Membranes and Decreases Sphingosine, Promoting Bacterial Infections. *Cell Host Microbe* 2017; 21: 707-718 e708.

E7. Snouwaert JN, Brigman KK, Latour AM, Malouf NN, Boucher RC, Smithies O, Koller BH. An animal model for cystic fibrosis made by gene targeting. *Science* 1992; 257: 1083-1088.

E8. Zhou L, Dey CR, Wert SE, DuVall MD, Frizzell RA, Whitsett JA. Correction of lethal intestinal defect in a mouse model of cystic fibrosis by human CFTR. *Science* 1994; 266: 1705-1708.

E9. Teichgraber V, Ulrich M, Endlich N, Riethmuller J, Wilker B, De Oliveira-Munding CC, van Heeckeren AM, Barr ML, von Kurthy G, Schmid KW, Weller M, Tummler B, Lang F, Grassme H, Doring G, Gulbins E. Ceramide accumulation mediates inflammation, cell death and infection susceptibility in cystic fibrosis. *Nat Med* 2008; 14: 382-391.

E10. Dorin JR, Dickinson P, Alton EW, Smith SN, Geddes DM, Stevenson BJ, Kimber WL, Fleming S, Clarke AR, Hooper ML, et al. Cystic fibrosis in the mouse by targeted insertional mutagenesis. *Nature* 1992; 359: 211-215.

E11. Nicklas W, Baneux P, Boot R, Decelle T, Deeny AA, Fumanelli M, Illgen-Wilcke B, Felasa. Recommendations for the health monitoring of rodent and rabbit colonies in breeding and experimental units. *Lab Anim* 2002; 36: 20-42.
